# Operando XANES Reveals the Chemical State of Iron‐Oxide Monolayers During Low‐Temperature CO Oxidation

**DOI:** 10.1002/cphc.202400835

**Published:** 2024-11-20

**Authors:** Dorotea Gajdek, Harald J. Wallander, Giuseppe Abbondanza, Gary S. Harlow, Johan Gustafson, Sara Blomberg, Per‐Anders Carlsson, Justus Just, Edvin Lundgren, Lindsay R. Merte

**Affiliations:** ^1^ Department of Materials Science and Applied Mathematics Malmö University SE-205 06 Malmö Sweden; ^2^ NanoLund Lund University Box 118 SE-221 00 Lund Sweden; ^3^ Division of Synchrotron Radiation Research Lund University Box 118, S E-221 00 Lund Sweden; ^4^ Department of Chemical Physics Chalmers University of Technology SE-412 96 Göteborg Sweden; ^5^ Department of Chemistry and Biochemistry and the Oregon Center for Electrochemistry University of Oregon Eugene Oregon 97403 United States; ^6^ Department of Process and Life Science Engineering Lund University Box 118 SE-221 00 Lund Sweden; ^7^ Department of Chemistry and Chemical Engineering Chalmers University of Technology SE-412 96 Göteborg Sweden; ^8^ Competence Centre for Catalysis Chalmers University of Technology SE-412 96 Göteborg Sweden; ^9^ MAX IV Laboratory Lund University Box 118 SE-221 00 Lund Sweden

**Keywords:** Heterogeneous catalysis, Iron oxide, Operando, Thin films, XAFS

## Abstract

We have used grazing incidence X‐ray absorption near edge spectroscopy (XANES) to investigate the behavior of monolayer FeO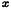 films on Pt(111) under near ambient pressure CO oxidation conditions with a total gas pressure of 1 bar. Spectra indicate reversible changes during oxidation and reduction by O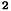 and CO at 150 °C, attributed to a transformation between FeO bilayer and FeO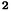 trilayer phases. The trilayer phase is also reduced upon heating in CO+O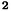 , consistent with a Mars‐van‐Krevelen type mechanism for CO oxidation. At higher temperatures, the monolayer film dewets the surface, resulting in a loss of the observed reducibility. A similar iron oxide film prepared on Au(111) shows little sign of reduction or oxidation under the same conditions. The results highlight the unique properties of monolayer FeO and the importance of the Pt support in this reaction. The study furthermore demonstrates the power of grazing‐incidence XAFS for in situ studies of these model catalysts under realistic conditions.

## Graphical abstract

**Follow the changes**! Operando time‐resolved GI‐XANES was used to follow structural changes of sub‐monolayer FeO_*x*_/Pt(111) during exposure to CO and O_2_ at ambient pressure conditions. We present experimental insight into the reaction mechanism: reduction of the trilayer phase upon heating in CO+O_2_ is consistent with a Mars‐van Krevelen type mechanism thus supporting the suggestion that the trilayer phase is the source of oxygen in CO oxidation.

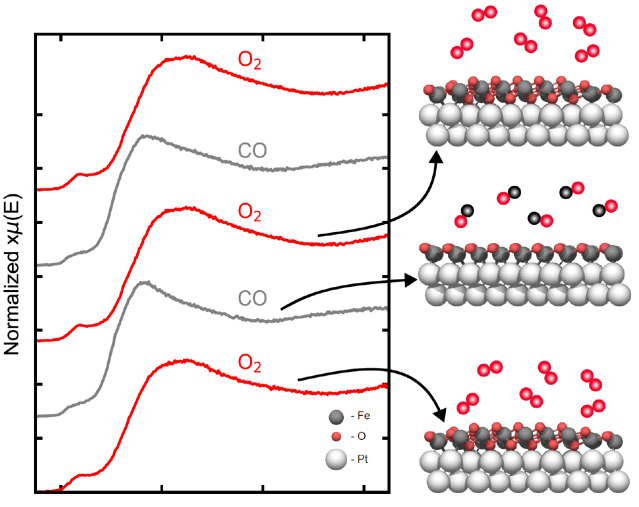

## Introduction

Ultra‐thin oxide films on metal supports are interesting systems for the study of metal/oxide interfaces,[^1^, ^2^, ^3^, ^4^] and several have been shown to exhibit enhanced catalytic activity, particularly for low temperature CO oxidation[^5^, ^6^] and the water gas shift reaction.^[7]^ Such wetting oxide layers are therefore believed to be important in the function of catalysts for e. g. hydrogen purification by CO preferential oxidation.^[8]^

**Figure 1:**
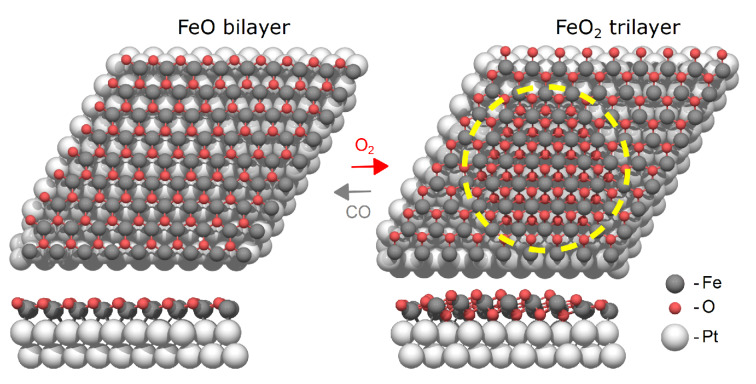
Models of the FeO_*x*_/Pt(111) phases expected from previous experiments. a) FeO bilayer supported on Pt(111), with oxygen atoms buckled slightly away from the surface. b) FeO_2_ trilayer (indicated by a yellow circle) supported on Pt(111), with an additional oxygen intercalated between the iron and the platinum.

Monolayers of FeO on Pt(111) are particularly well‐studied. These layers exhibit a hexagonal boron nitride‐type structure with oxygen atoms buckled slightly away from the surface,[^9^, ^10^, ^11^, ^12^] as illustrated in Figure 1. We refer to this phase as the “bilayer”, in reference to the single layers of Fe and O. As demonstrated first by Sun et al.,[^5^, ^13^] these oxides show activity towards CO oxidation at around 450 K, a temperature at which Pt(111) itself is not catalytically active due to adsorbed CO blocking the sites required for O_2_ dissociation. They showed that under oxygen‐rich conditions, the FeO film is converted to a trilayer FeO_2_ phase where oxygen is intercalated between the iron and the platinum (Figure 1). Subsequent CO exposure reduces this back to the bilayer phase.[^14^, ^15^]

Low‐temperature CO oxidation activity has indeed been observed for iron oxide monolayers on Pt(111) and attributed to this transformation.[^5^, ^13^, ^14^, ^15^, ^16^, ^17^] The reversible transformation between bilayer FeO and trilayer FeO_2_ makes possible a Mars‐van‐Krevelen type mechanism for CO oxidation that avoids competitive adsorption on the Pt surface and allows the reaction to proceed at low temperatures. The formation of the trilayer phase and its involvement in the catalytic reaction would appear to depend on the reaction conditions, with highly reducing conditions tending to keep the oxide in a more reduced state. Bilayer FeO islands can themselves exhibit low‐temperature CO oxidation activity via reactions at island edges,[^6^, ^18^] and alloying of iron with Pt has been shown to modify the chemisorption behavior of the noble metal.[^19^, ^20^]

To probe these processes and identify the relevant phases formed by the iron promoter, it is necessary to characterize the model catalysts under in situ or operando conditions, corresponding to a gas environment at atmospheric pressure or above or to a liquid electrolyte. Such conditions pose significant challenges for surface studies due to the scattering of electrons and photons by the gas or the liquid.

X‐ray absorption spectroscopy in the hard X‐ray range can be applied to sub‐monolayer oxides on metal single crystals with grazing incidence geometry and fluorescence detection, and was shown recently to be a useful tool to study ultra thin cobalt oxides under ambient pressure conditions.^[21]^ In this previous experiment, XAFS spectra were acquired at different temperatures in a stepwise manner, with data collection taking around 1 h at each point. Although this allowed for changes to be followed under different conditions, for operando measurements where dynamic changes can be expected, more rapid data acquisition is highly desirable.

By focusing on the near‐edge region exclusively and making use of the high performance Balder beamline[^22^, ^23^] at the MAX IV Laboratory, we are able to reduce the acquisition time per spectrum to about 20s, allowing for measurements to be acquired during temperature ramps. The results thus demonstrate how XANES measurements can be applied to follow the chemical state of such atomically thin oxides under changing conditions.

Here, we present XANES measurements of sub‐monolayer FeO on Pt(111) at the Fe K‐edge (7112 eV) under atmospheric pressure CO oxidation conditions. We demonstrate oxidation and reduction of the monolayer in the single gases as well as during heating in a CO+O_2_ mixture. For comparison and to shed light on the role of the support metal, we also grew and characterized a monolayer iron oxide on Au(111), which was reported to exhibit a similar structure to the film on Pt(111) support.^[24]^

## Results

**Figure 2:**
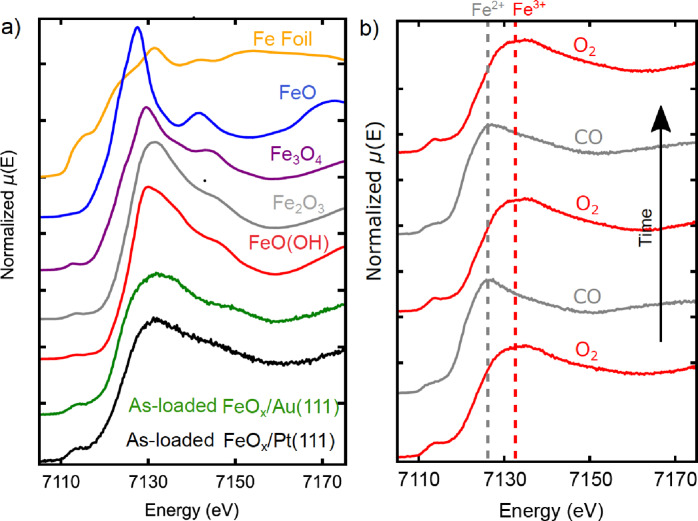
a) XANES spectra of the as‐loaded FeO_*x*_/Pt(111) and FeO_*x*_/Au(111) together with reference spectra of Fe metal, FeO(OH), Fe_2_O_3_, Fe_3_O_4_ and FeO. b) XANES spectra acquired at 150 °C on the monolayer FeO_*x*_/Pt(111) sample under CO and O_2_, showing features characteristic of Fe^2+^ and Fe^3+^, as expected. The arrow indicates the order in which the measurements were taken.

XANES spectra of the Au and Pt‐supported iron oxide samples in the as‐loaded state, recorded under helium at room temperature, are shown in Figure 2a, along with reference spectra for several bulk iron compounds. In both cases the spectra appear to indicate iron in the 3+ oxidation state, showing similarity to both hematite (Fe_2_O_3_) and goethite (FeO(OH)). We expect that during the transfer to the cell via the glove box, the bilayer samples were exposed to O_2_ and H_2_O with considerable concentrations, even it was in the sub‐ppm atmosphere. Given that formation of an oxyhydroxide is observed upon oxidation even under ultra‐clean vacuum conditions,[^25^, ^26^] we expect this (monolayer FeO(OH)) is the state of our samples at the start of our experiments.

To investigate the oxidation and reduction behavior of the FeO_*x*_/Pt(111), we began by measuring XANES spectra at increasing temperatures, alternating the gas between O_2_ and CO (each 50 mbar in He) at each step. Significant changes were first observed during exposures at 150 °C, which is comparable to the temperatures at which the formation of a FeO_2_ trilayer was reported in previous studies.[^5^, ^14^, ^20^]

XANES spectra recorded at this temperature are plotted in Figure 2b, and show clear changes in the spectra in response to the gas. These changes appear to correspond to a transformation between Fe^3+^ and Fe^2+^ states, as expected for the bilayer/trilayer transition.^[26]^

**Figure 3:**
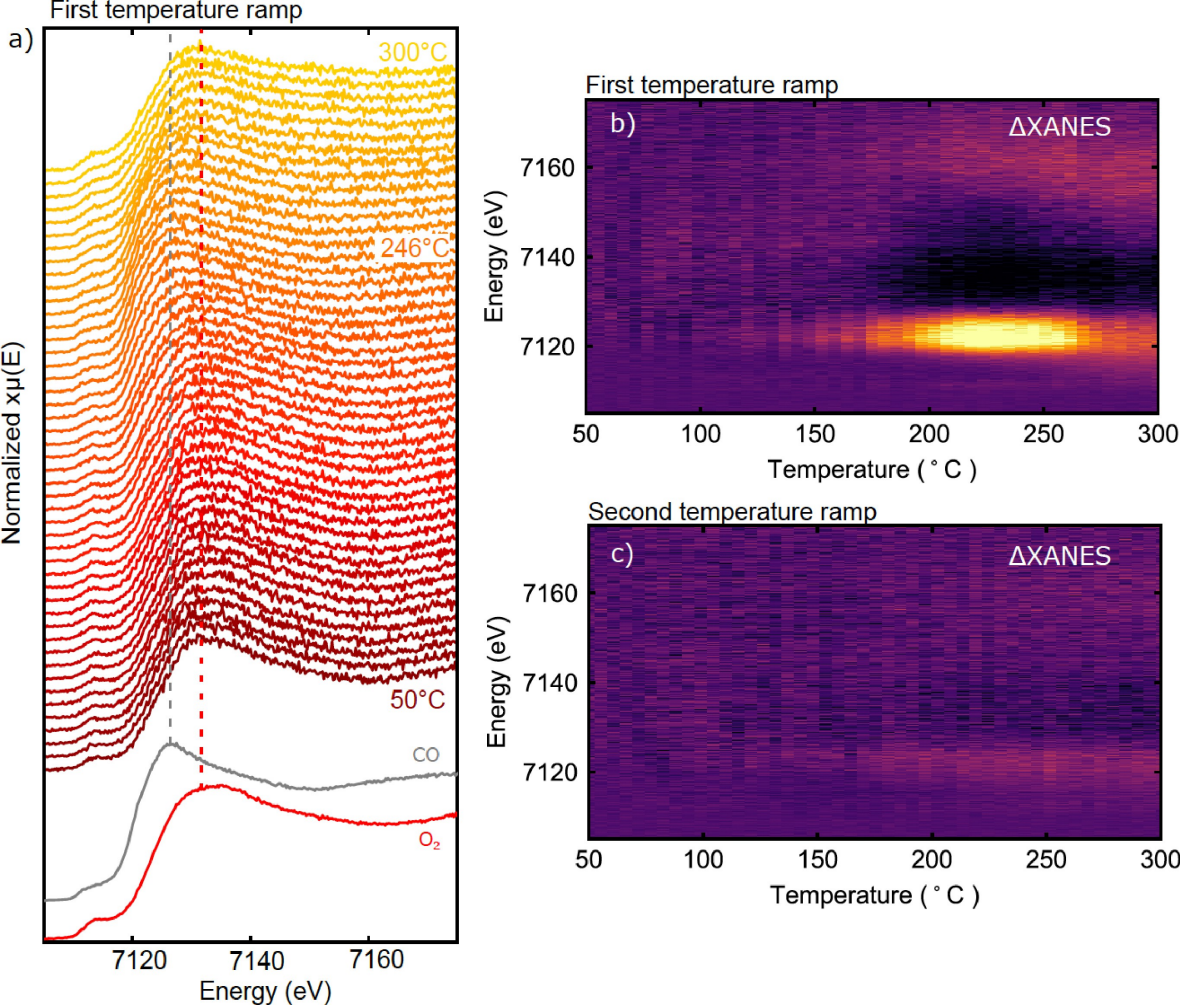
Summary of operando XANES measurements from FeO_*x*_/Pt(111) during CO oxidation in 1 : 1 CO:O_2_, with a 25 mbar partial pressure for each gas. a) Individual scans acquired during the temperature ramp from 50–300 °C. Spectra corresponding to the reduced and oxidized states formed under CO and O_2_, respectively, at 150 °C are shown for reference. b) Difference plot showing ΔXANES for the spectra in a), with an averaged spectrum at the start of the experiment taken as reference. Reduction of the oxide is evident from the bright feature at ∼7125 eV, with an onset at about 150 °C. c) Corresponding ΔXANES plot for the second ramp (individual scans shown in Figure S2a in the Supplementary Information).

After the oxidation and reduction tests, the sample was tested under CO oxidation conditions. After the final oxidation step at 150 °C, the sample was cooled in O_2_ to 50 °C and CO was introduced to form a 1 : 1 mixture of both (25 mbar partial pressure of each). The temperature was then ramped from 50 °C to 300 °C with simultaneous spectra collection, at a rate of 10 °C/min. The XANES spectra acquired during this first temperature ramp are shown in Figure 3a.

The first peak above the edge is typically the most intense absorption feature and is very sensitive to the chemical environment of iron. Here we refer to this peak as the “white line” of the spectrum. Initially, the peak is seen at ~7132 eV, corresponding to the Fe^3+^ state observed at 150 °C. With increasing temperature, a shift in the white line towards lower energies is observed, indicating reduction of the film. For easier visualization of the changes to the spectra, a difference plot is shown in Figure 3b, where the average of 5 spectra at the start of the experiment is taken as a reference. The shift of the spectrum to lower energy is observed here as the bright feature at ~7128 eV. The onset of the transformation is observed around 150 °C, and reaches a maximum extent around ~240 °C. At higher temperatures, the spectrum shifts again to higher energy, indicating re‐oxidation of the film.

After the first temperature ramp, the sample was cooled to 50 °C in the same gas mixture and the experiment was repeated. XANES spectra from the second ramp (Figure 3c) show only very minor changes with temperature, indicating that the transformation observed above 250 °C in the first ramp was irreversible.

Linear combination analysis (LCA) was performed for these data sets using spectra collected under pure O_2_ and pure CO at 150 °C as references (see Supplementary Information). These components describe the spectra up to ~220 °C well, but were insufficient to fit the data at the highest temperatures where the shift to higher energy was observed; in particular, an increased intensity in the pre‐edge range is observed which cannot be accounted for by the oxide phases. Inclusion of reference spectra from bulk oxides/hydroxides also did not improve the fits. Adequate fits could, however, be obtained by inclusion of a metallic component based on Fe foil, suggesting that the formation of Fe^3+^ is accompanied by partial reduction to Fe metal.

**Figure 4:**
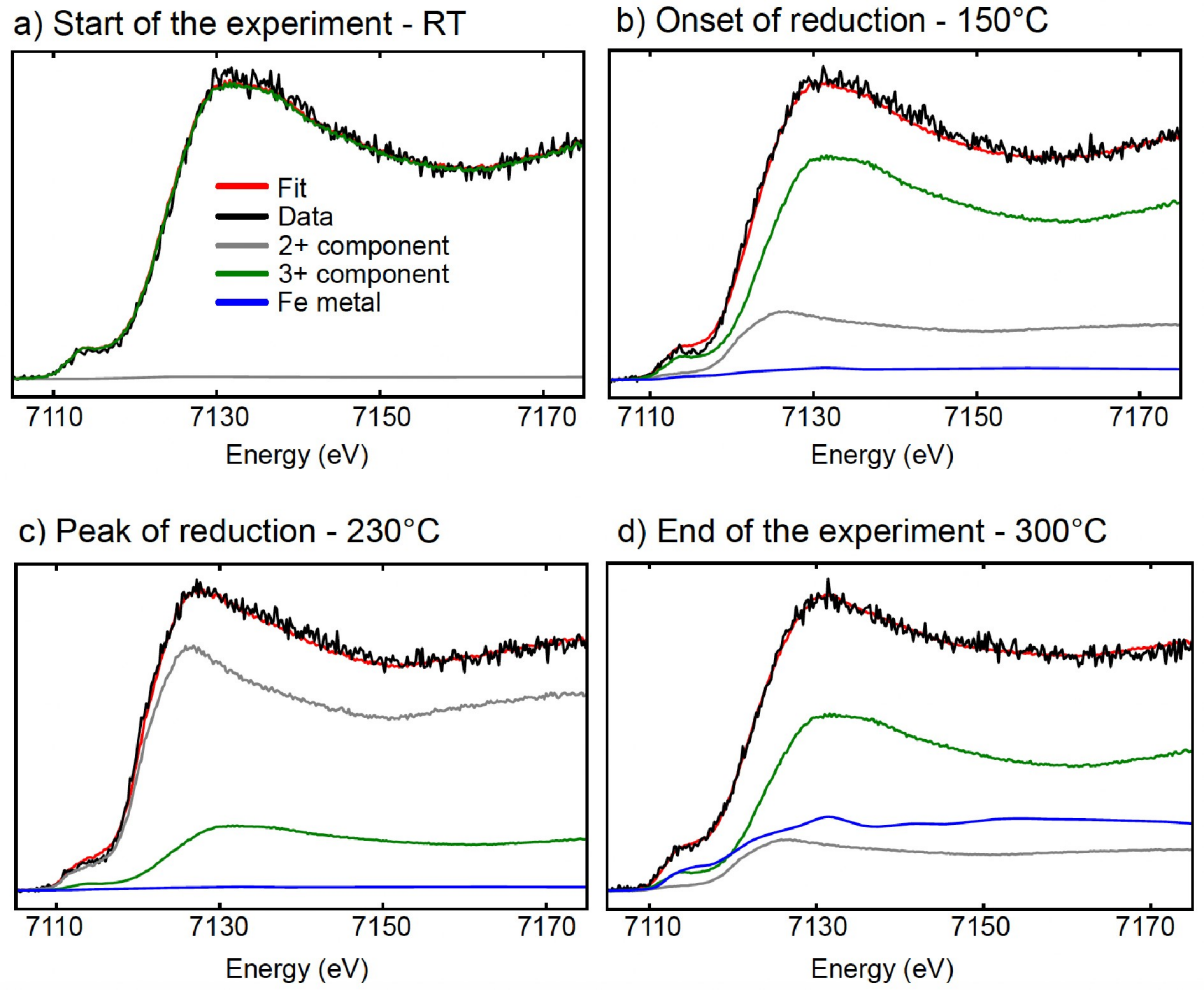
Linear combination analysis fits for the first temperature ramp over FeO_*x*_/Pt(111). a) Fitted spectra at the start of the experiment. b) Fitted spectra at the onset of reduction. c) Fitted spectra at the peak of reduction. d) Fitted spectra at the end of the experiment.

**Figure 5:**
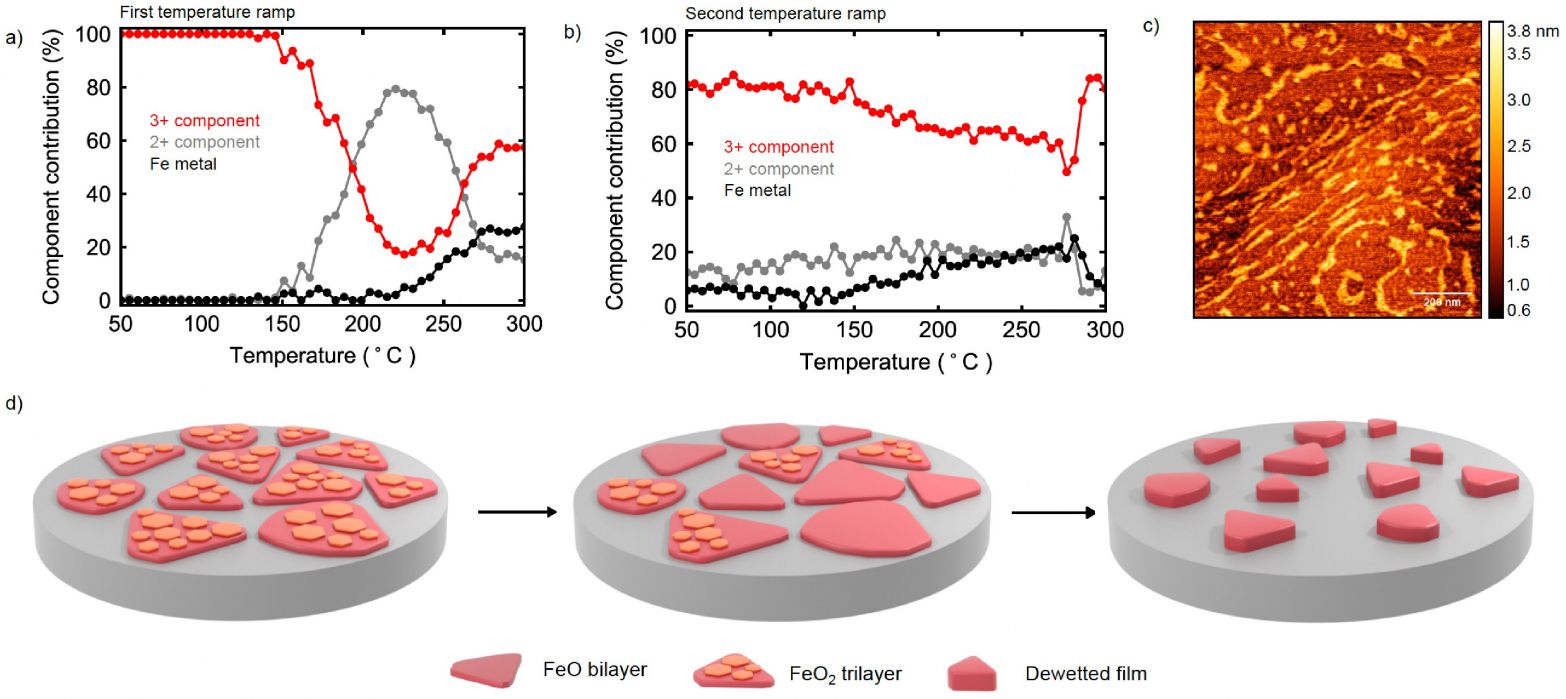
a) Component contribution during the first temperature ramp over FeO_*x*_/Pt(111) extracted using linear combination fitting. b) Component contribution during the second temperature ramp over spent FeO_*x*_/Pt(111) extracted using linear combination fitting. c) Post mortem AFM of the FeO_*x*_/Pt(111) after the second temperature ramp showing dewetting of the film. d) Models depicting our interpretation of the XANES data acquired during the first temperature ramp. The reduction of the trilayer phase is followed by dewetting of the film.

Figure 4 shows LCA fits for selected spectra from the first temperature ramp. The resulting component fractions are shown in Figure 5a. Here, we can see the initial state of iron in the film to be the fully oxidized 3+ state. At ~150 °C we observe a loss of the 3+ component and increase of the 2+ component, consistent with the shift described above. Reappearance of the 3+ component occurs above ~230 °C. The results of similar analysis for the second temperature ramp are shown in Figure 5b. Fits of selected spectra from this data set are presented in the Supplementary Information.

The drastic change in the reduction behavior of the iron following the first temperature ramp indicates that a significant change in the film occurred. *Post mortem* AFM analysis (Figure 5c) showed islands ~2 nm in thickness, corresponding to several atomic layers of FeO. This result indicates that the monolayer phase has dewetted the Pt surface. So‐called “reactive” dewetting of FeO monolayers under CO+O_2_ – and involving the interaction with both gases – was reported by Sun et al.[^5^, ^13^] The appearance of an apparent metallic component in the XANES spectra in addition to the oxide components suggests that this dewetting process involves both a reduction by CO and an oxidation by O_2_, either in sequence or in parallel.

Figure 5d shows graphical models of the FeO_*x*_ surface on Pt(111) before, during and at the end of the first temperature ramp, representing our interpretation of the XANES data and the *post mortem* AFM analysis. The first transformation observed corresponds to the reduction of the FeO_2_ trilayer to the FeO bilayer, with oxygen removal significantly outpacing reoxidation by O_2_ above 200 °C. The second transformation is the reactive dewetting to form thicker iron oxide islands with a minority fraction of metallic iron.

**Figure 6:**
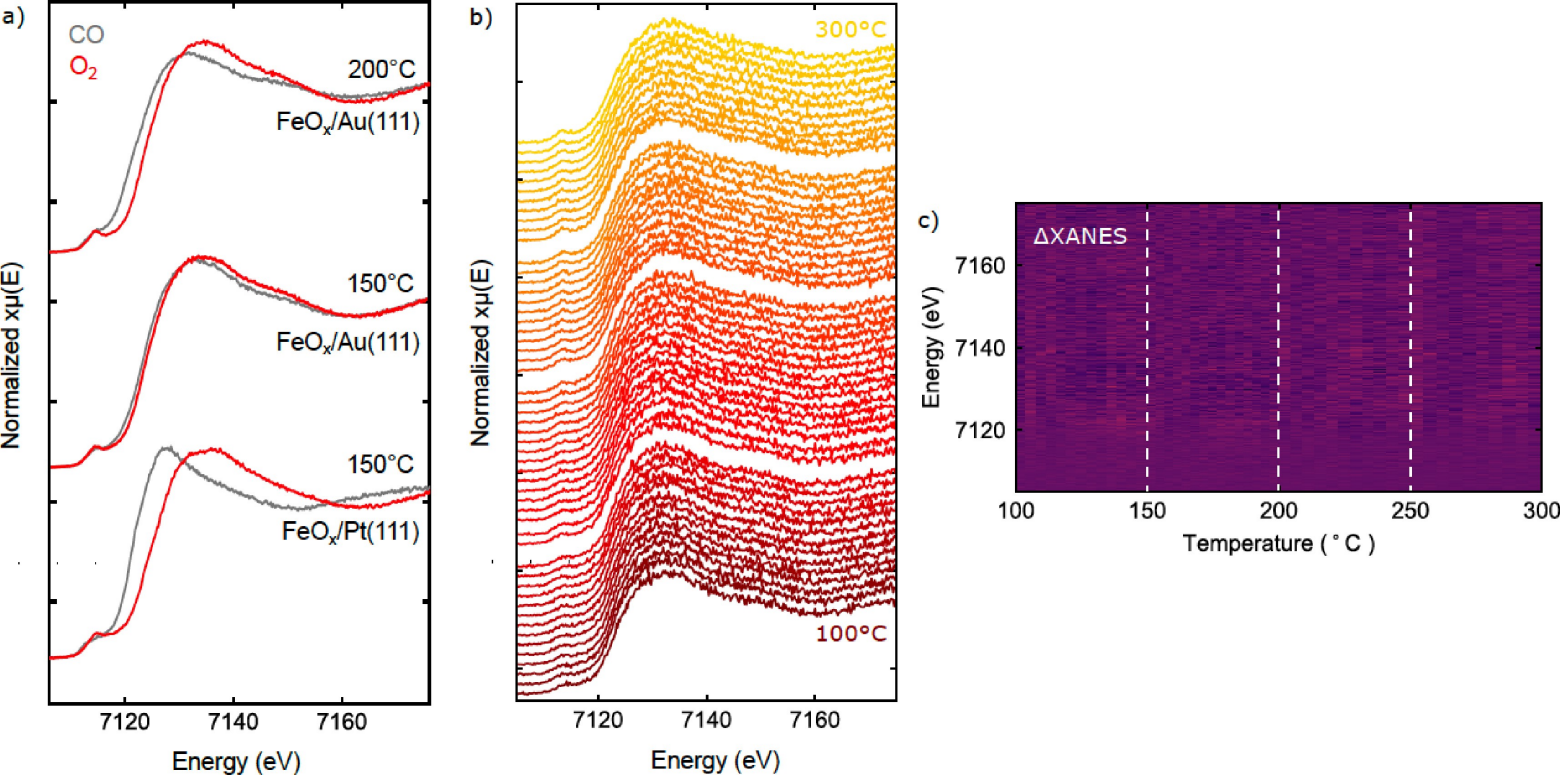
a) XANES spectra measured in alternating pure CO and O_2_ gases for FeO_*x*_/Au(111), with the spectra from FeO_*x*_/Pt(111) at 150 °C for comparison. b) Individual scans acquired during the temperature ramp from 50 °C–300 °C over FeO_*x*_/Au(111). c) Difference plot showing ΔXANES for the spectra in b), with an average of spectra at the start of the experiment taken as reference. No changes in the spectra were observed during the temperature ramps.

In order to investigate the effect of the support on the reactivity of the FeO_*x*_ film, we performed similar oxidation and reduction experiments for an FeO film grown on Au(111). Figure 6a shows XANES measurements in pure CO and O_2_ gases. We can see that at 150 °C, the film on Au(111) substrate showed little to no response to the gas changes. Temperature increase to 200 °C resulted in some reduction of the film, but to a smaller extent than on Pt(111) support. Further temperature increase on Au(111) support resulted in a higher degree of reduction, however, the film never reached the reduction level of that one on Pt(111).

The behavior of the FeO_*x*_/Au(111) sample in CO+O_2_ mixture was tested in between the measurements in pure gases during temperature ramps. The corresponding spectra from in situ measurements are shown in Figure 6b and a difference plot is shown in Figure 6c. The measurements were acquired during the 50 °C temperature ramps between 100 °C and 300 °C. Consistent with the smaller response of the iron to the pure gases, we did not observe any structural changes with temperature in the CO+O_2_ gas mixture.

The gases exiting the flow cell were sampled and analyzed with a quadrupole mass spectrometer. These data showed negligible conversion of CO during experiments with the FeO_*x*_/Au(111) sample, and CO conversion typical of platinum for the experiments with FeO_*x*_/Pt(111). Although some differences in conversion were observed during the first temperature ramp over FeO_*x*_/Pt(111) compared with the second, these could not be reliably distinguished from drift in the measured CO_2_ signal. The small observed low‐temperature conversion is attributed to low sensitivity under our flow conditions and to the small density of edge sites for our oxide islands.

## Discussion

Our experiments on FeO_*x*_/Pt(111) during the exposure to pure CO and O_2_ gases at 150 °C showed the films to react strongly in response to the gases, reflected in the transformations between 3+ and 2+ states of iron. This reversibility of the oxidation and reduction processes at low temperatures is consistent with the catalytic mechanism proposed by Sun et al. In particular, the facile reduction of the oxidized phase by CO indicates availability of oxygen from the trilayer for the production of CO_2_, which can be understood as a Mars‐van Krevelen (MvK) mechanism with the 2D iron oxide serving as an oxygen reservoir.

Time resolved XANES spectra acquired in the CO oxidation experiments under ambient pressure reaction conditions were found to be of good quality and showed high sensitivity to structural changes of submonolayer oxides. To our knowledge, this was the first in situ observation of FeO_2_ trilayer reduction to FeO bilayer under ambient‐pressure CO oxidation conditions. Although the data do not provide proof that the MvK mechanism is the dominant process in the low‐temperature CO oxidation reaction, the reduction of the trilayer upon the temperature increase in CO+O_2_ does indicate that this reaction takes place at a significant rate and that the mechanism could contribute under conditions that are sufficiently oxidizing for the trilayer to form.[^5^, ^13^, ^14^, ^15^] The behavior observed in the second temperature ramp, explained by the already mentioned dewetting of the film, further demonstrates the unique role of the monolayer FeO film in promoting catalytic activity for the CO oxidation reaction.

Our results, measured at 1 bar total pressure, highlight the value of GI‐XAFS in studies of reaction mechanisms and structural changes of ultra‐thin oxides at realistic reaction conditions. XANES energy scans were quick enough to follow structural changes in situ due to the high signal‐to‐noise ratio, which was achieved using an energy discriminating detector. On the other hand, it was not possible to measure in situ EXAFS due to the longer scan time, which prevented observations of structural changes in situ. The cell used a graphite dome, which allowed for measurements of sub‐monolayer iron oxide films. In a previous attempt at this experiment, the iron contribution to the fluorescence signal from the beryllium dome used in the first version of our cell^[21]^ was larger than that from the sample. However, the cell holds a high volume of gas, which negatively affects the sensitivity of our experiment for catalysis. The high gas flow, used to reduce possible contaminants on the surface, further added to the reduced sensitivity. In the future, reducing the gas flow and the volume of the cell should provide good enough conditions to detect the catalytic activity during the reaction.

A source of uncertainty in these experiments remains the effect of the gas exposure during sample transfer from the UHV preparation chamber to the catalytic reaction cell. Even though we transferred the samples in the vacuum suitcase and a static vacuum chamber, the loading of the sample in the cell was done in the glove box, whose low concentrations of O_2_ and H_2_O were enough to alter the deposited structure of our films. Although this is not expected to change them drastically – the monolayer morphology is stable under gas exposure at room temperature – the reactions with oxygen and water prevent use of the as‐prepared sample as a reference for the bilayer phase, for example. For future investigations, a full transfer in vacuum would be desirable to maintain the films in their as‐prepared states.

The effect of the support was investigated by growing the FeO_*x*_ film on Au(111) substrate, since it was shown that the structure strongly resembles that of FeO_*x*_/Pt(111).^[27]^ The spectrum recorded after transfer resembles that of the as‐loaded FeO_*x*_/Pt(111) sample, which we interpret as a similar oxyhydroxide phase. A hydroxylated structure like this was previously^[25]^ found to form on FeO_*x*_/Au(111) under water exposure, similarly as for the FeO_*x*_/Pt(111). Spectra resembling either the bilayer or trilayer phases on Pt(111) were not observed, however, which we suspect mainly reflects the important role of the Pt surface in mediating oxidation and reduction reactions. The *post mortem* AFM analysis (see Supplementary Information) showed intact FeO_*x*_ islands embedded in the Au(111) surface, which was previously observed when annealing such films to high temperatures. It is not clear at which temperature this embedding took place, but if this morphology causes the oxide island edge sites to be blocked, this would contribute additionally to the reduced reactivity of the films.

## Conclusions

In summary, we have shown the reversibility of the changes between Fe^3+^ and Fe^2+^ components in FeO_x_/Pt(111) during exposure to O_2_ and CO, respectively, which is consistent with the reported activity of the FeO_*x*_/Pt(111) in catalytic CO oxidation. Testing the film in operando CO oxidation conditions showed that oxidized FeO film exhibits structural changes and therefore activity in the reaction. This was manifested by the reduction of the film with the temperature increase, specifically around 150 °C where it was previously shown that the transition between two FeO structures occurs. Further oxidation of the film at higher temperatures supports the operation of a Mars‐van Krevelen (MvK)‐type mechanism as the film is restored to a trilayer during a reaction with O_2_, although it remains unclear whether the steady‐state rates of the reaction are high enough to explain the observed low‐temperature reactivity. The loss of activity during the second ramp is attributed to the dewetting of the film which was observed in *post mortem* AFM analysis and supports the notion that the iron oxide monolayer and not iron oxides more generally – is key to the promoting effect of iron.

Despite having a similar structure, the FeO_*x*_ film supported on Au(111) does not show similar changes during the exposure to O_2_ and CO under the same conditions. The lack of reactivity in the oxidation/reduction studies over gold support highlights the importance of the platinum substrate in low temperature CO oxidation promoted by iron oxide monolayers.

## Experimental

### Experimental Methods

**Growth of FeO_x_ thin films**. Samples were prepared in an ultra‐high vacuum (UHV) molecular beam epitaxy (MBE) system with a base pressure of $1\times 10^{-10}$ mbar. Pt(111) (Surface Preparation Laboratory, NL) was cleaned by cycles of Ar^+^ sputtering followed by annealing in $5\times 10^{-7}$ mbar O_2_ at 600 °C for 5 min to remove carbon contamination, followed by brief heating in vacuum to 800 °C to desorb oxygen. Au(111) (Surface Preparation Laboratory, NL) was cleaned by Ar^+^ sputtering followed by annealing in UHV at 570 °C for 15 min. The sample temperature was estimated using a C‐type thermocouple mounted at the heater stage, which was calibrated initially against a K‐type thermocouple welded to the side of the crystal, which was later removed to allow for sample transport.

The FeO_x_ films with coverage of about 0.5 ML were grown by electron‐beam evaporation (Specs EBE‐4) from a 2 mm Fe rod (99.999 %, Mateck) using a multi‐pocket evaporator. The deposition rate was calibrated with a quartz crystal microbalance (QCM). FeO/Pt(111) was prepared by deposition in $5\times 10^{-7}$ mbar O_2_ at room temperature (RT) followed by heating at 600 °C for 3 min in the same atmosphere. FeO/Au(111) was prepared following Yang et al.^[24]^ by deposition of metallic iron at room temperature followed by post‐oxidation in $1\times 10^{-6}$ mbar O_2_ at RT and then vacuum annealing at 500 °C. The desired film structure was confirmed in situ by reflection high energy electron diffraction (RHEED) and further confirmed by low energy electron diffraction (LEED) measured in a separate system after transfer in a UHV vacuum suitcase.

**GI‐XAFS measurements**. Fe K‐edge (E_0_=7112 eV) GI‐XAFS spectra were recorded at the Balder beamline[^22^, ^23^] at the MAX IV Laboratory, Lund, Sweden. XANES spectra were recorded in grazing incidence (0.5°) with out‐of‐plane polarization. Absorption spectra were acquired using the Fe‐K*α* fluorescence signal from a seven‐element germanium detector. Spectra acquired under static conditions were recorded from E_0_‐60 eV to E_0_+200 eV. Spectra acquired during heating ramps ranged from E_0_‐10 eV to E_0_+70 eV with a repetition rate of about 20 s per spectrum. XANES data processing and analysis made use of the Larch^[28]^ package. Linear combination analysis was performed using Fe foil measurement for Fe^0^ state and the state of the film under pure O_2_ and CO flows at 150 °C for Fe^3+^ and Fe^2+^ states, respectively. A reference spectrum for Fe foil was measured at the beamline while spectra for FeO(OH), Fe_2_O_3_, Fe_3_O_4_ and FeO was taken from the Lytle Database.^[29]^

The sample was transferred from the UHV system to a vacuum suitcase with base pressure of $1\times 10^{-9}$ mbar. The sample was transferred to the beamline in the vacuum suitcase where it was transferred to a small vacuum chamber via pumping station. The vacuum chamber was then loaded into the glove box, together with the reaction cell. The reaction cell, described previously,^[21]^ is a flow cell based on a hemispherical, X‐ray transparent dome, with the sample fixed atop a graphite/boron‐nitride heater. The cell was mounted at the Balder end station with surface normal parallel to the beam polarization and an incident angle of about 0.5°. CO oxidation conditions were achieved with O_2_ and CO mixed in 1 : 1 ratio. Each of the gases was diluted to 5 % in He with the purity of at least N4.6. The total pressure during the in situ measurements was set to 1 bar, resulting in 25 mbar partial pressure for each gas. Total flow was set to 50 mL/min and controlled by individual mass flow controllers (Bronkhorst). Exhaust gas from the cell was sampled via an adjustable leak valve and its composition was measured using a quadruple mass spectrometer (Pfeiffer PrismaPlus). During time resolved measurements, the temperature was increased from room temperature (RT) to 300 °C with the rate of 10 °C/min.

## Conflict of Interests

The authors declare no conflict of interest.

1

## Supporting information

As a service to our authors and readers, this journal provides supporting information supplied by the authors. Such materials are peer reviewed and may be re‐organized for online delivery, but are not copy‐edited or typeset. Technical support issues arising from supporting information (other than missing files) should be addressed to the authors.

Supporting Information

## Data Availability

The data that support the findings of this study are available from the corresponding author upon reasonable request.
