# Supplementary material for: Operando XANES Reveals the Chemical State of Iron‐Oxide Monolayers During Low‐Temperature CO Oxidation
Source: Chemphyschem. 2024 Nov 20;26(2):e202400835. doi: 10.1002/cphc.202400835 (PMC11733406; doi:10.1002/cphc.202400835)
Supplement: Supplementary file 1 — Supporting Information [file CPHC-26-e202400835-s001.pdf]

ChemPhysChem

Supporting Information

Operando XANES Reveals the Chemical State of Iron-Oxide Monolayers During Low-Temperature CO Oxidation

Dorotea Gajdek, Harald J. Wallander, Giuseppe Abbondanza, Gary S. Harlow, Johan Gustafson, Sara Blomberg, Per-Anders Carlsson, Justus Just, Edvin Lundgren, and Lindsay R. Merte*

Supplementary Information

Dorotea Gajdek,[†,‡] Harald J. Wallander,[†,‡] Giuseppe Abbondanza,[¶] Gary S. Harlow,[§] Johan Gustafson,[‖] Sara Blomberg,[⊥,‡] Per-Anders Carlsson,[#,@] Justus Just,[△] Edvin Lundgren,[‖,‡] and Lindsay R. Merte[*,†,‡]

†*Department of Materials Science and Applied Mathematics, Malmö University, SE-205 06 Malmö, Sweden*

‡*NanoLund, Lund University, Box 118, SE-221 00 Lund, Sweden*

¶*Department of Chemical Physics, Chalmers University of Technology, SE-412 96 Göteborg, Sweden*

§*Department of Chemistry and Biochemistry and the Oregon Center for Electrochemistry, University of Oregon, Eugene, Oregon 97403, United States*

‖*Division of Synchrotron Radiation Research, Lund University, Box 118, SE-221 00 Lund, Sweden*

⊥*Department of Process and Life Science Engineering, Lund University, Box 118, SE-221 00 Lund, Sweden*

#*Department of Chemistry and Chemical Engineering, Chalmers University of Technology, SE-412 96 Göteborg, Sweden*

@*Competence Centre for Catalysis, Chalmers University of Technology, SE-412 96 Göteborg, Sweden*

△*MAX IV Laboratory, Lund University, Box 118, SE-221 00 Lund, Sweden*

E-mail: lindsay.merte@mau.se

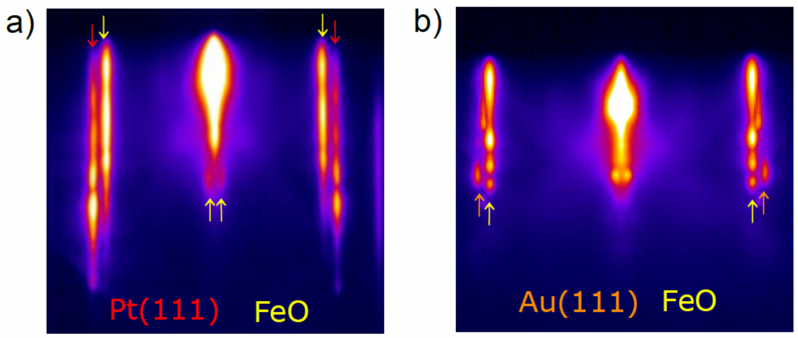

Figure S1: a) RHEED measurement of the as-deposited FeOx film on Pt(111) along Pt[11] direction. b) RHEED measurement of the as-deposited FeOx film on Au(111) along Au[11] direction

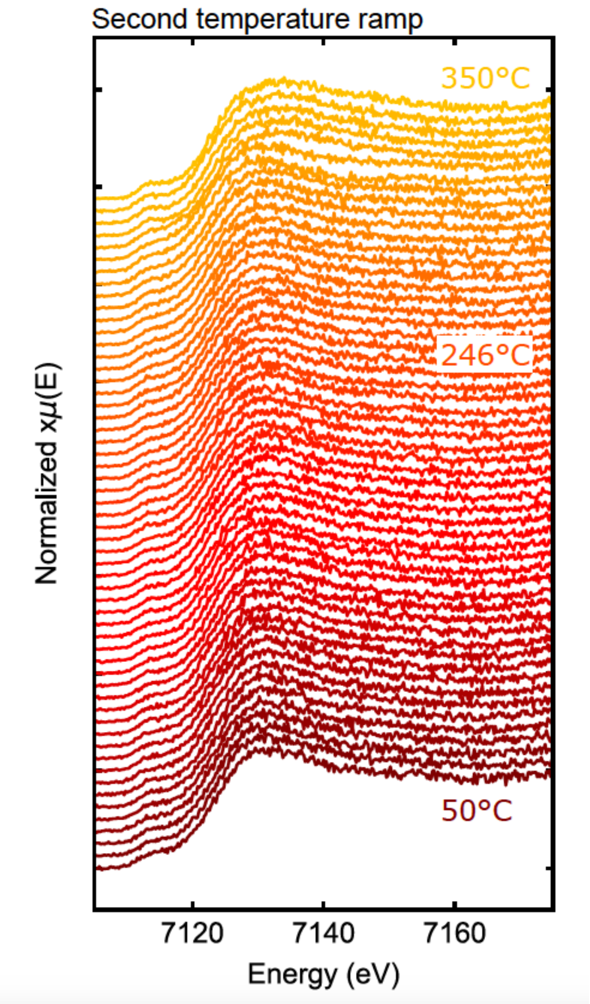

Figure S2: Summary of operando XANES measurements from FeO_x/Pt(111) during CO oxidation. Individual scans acquired during the second temperature ramp from 50 °C-300 °C on the spent sample.

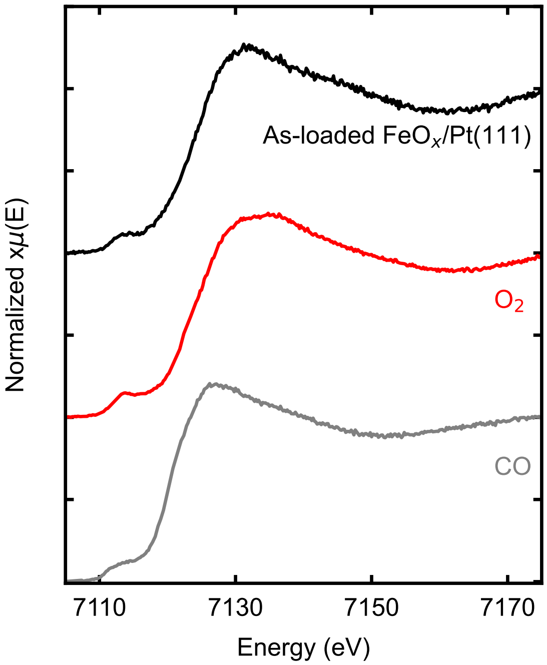

Figure S3: XANES spectra of the as-loaded FeO_x film on Pt(111), black. XANES spectra recorded at 150 °C in pure O_2 (red) and XANES spectra recorded at 150 °C in pure CO (gray). The spectra recorded in pure gases was used as a reference for Linear Combination Analysis (LCA).

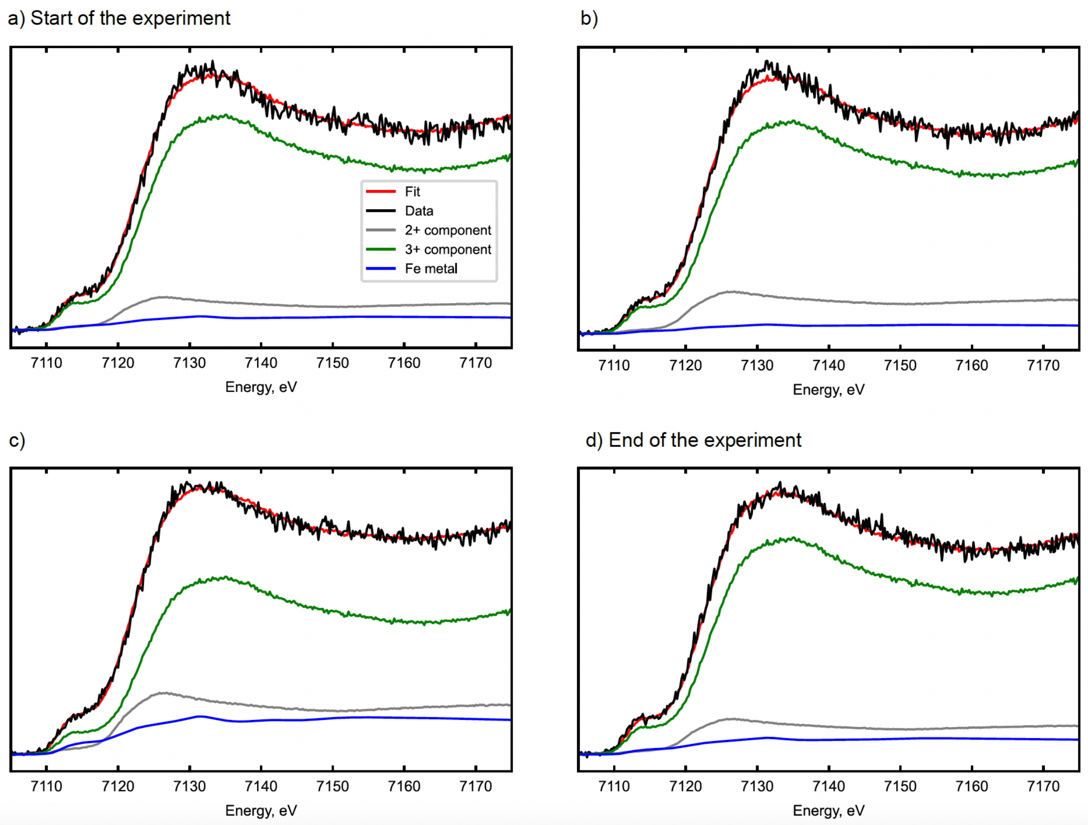

Figure S4: Linear combination analysis fits for the second temperature ramp over $FeO_x/Pt(111)$. a) Fitted spectra at the start of the experiment. b) Fitted spectra around 150 °C. c) Fitted spectra around 220 °C. d) Fitted spectra at the end of the experiment.

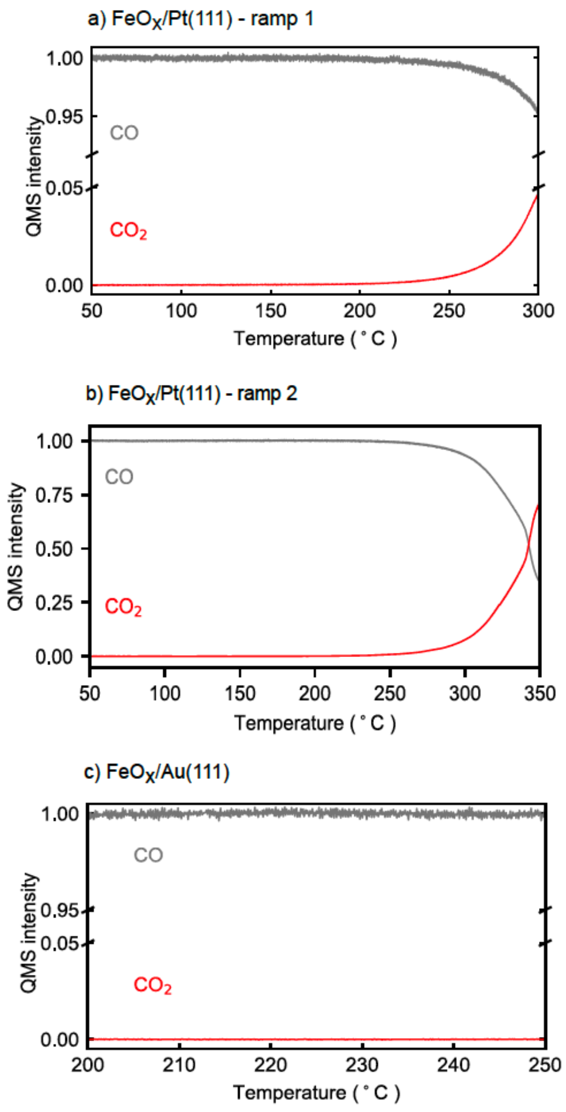

Figure S5: a) QMS data measured during the first temperature ramp over FeO_x/Pt(111). b) QMS data measured during the second temperature ramp over FeO_x/Pt(111). c) QMS data measured during the temperature ramp over FeO_x/Au(111). We show the range where we expect activity.

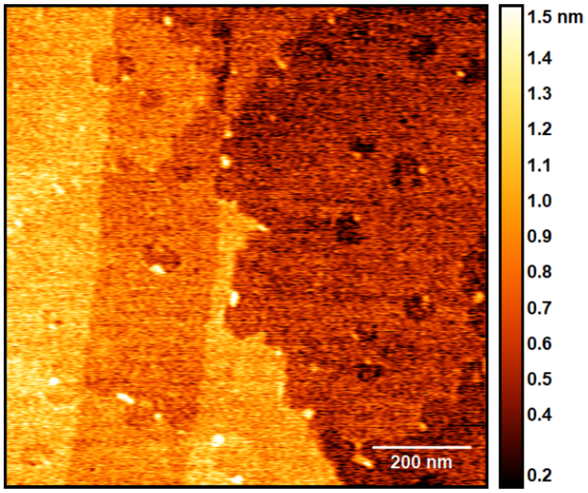

Figure S6: *Post mortem* AFM of the FeO_x/Au(111) showing embedded FeO_x islands in the Au(111) surface.
